# Coagulation/fibrinolysis and circulating tumor cells in patients with advanced breast cancer

**DOI:** 10.1007/s10549-021-06484-1

**Published:** 2022-02-08

**Authors:** Luc Y. Dirix, Steffi Oeyen, Andy Buys, Vincent Liégois, Annemie Prové, Tom Van De Mooter, Steven Van Laere, Peter B. Vermeulen

**Affiliations:** GZA Sint Augustinus, Oosterveldlaan 24, Wilrijk, 2610 Antwerp, Belgium

**Keywords:** Advanced breast cancer, Circulating tumor cells, D-dimers, Platelets, Diffuse intravascular coagulation, Tumor derived extracellular vesicles

## Abstract

**Purpose:**

To evaluate the relationship between circulating tumor cells (CTCs) and standard coagulation tests in both a discovery and a validation cohort of patients with advanced breast cancer.

**Methods:**

In a retrospective (*n* = 77) and a prospective (*n* = 92) study of patients with progressive advanced breast cancer, CTC count, platelet number, fibrinogen level, D-dimers, prothrombin time, and activated partial thromboplastin time were measured. The association between these coagulation studies and CTC count was analyzed. The impact of these measurements on overall survival (OS) was assessed.

**Results:**

In both cohorts, results were similar; absolute CTC count was significantly associated to D-dimer level and inversely with platelet count. In the prospective cohort, quantification of tumor-derived extracellular vesicles (tdEVs) was associated with CTC count, and with coagulation abnormalities (low platelet count and increased D-dimers). tdEVs did not add to CTC count in predicting changes in platelets or D-dimers. In multivariate analysis only CTC ≥ 5 CTC/7.5 mL, ER status, HER2 status and lines of chemotherapy were associated with OS. In patients with terminally metastatic breast cancer, very high CTC counts are prevalent.

**Conclusion:**

A significant association exists between increasing CTC number and increased D-dimers and decreased platelet counts, suggesting a potential role for CTCs as a direct contributor of intravascular coagulation activation. In patients with advanced and progressive breast cancer, abnormalities in routine coagulation tests is the rule. In patients with terminally advanced breast cancer a “leukemic” phase with high CTC count is prevalent.

## Introduction

Coagulation activation is detectable in plasma in the majority of patients with localized and/or advanced breast cancer [1–4]. Its biological origin and eventual clinical significance are less clear and might be underestimated. A relationship between markers of coagulation activation and prognosis has been established [5–7]. This activation might be considered solely as a bystander phenomenon of tumor growth, and eventually serve as a surrogate for disease burden, c.q. disease kinetics. There are however preclinical and clinical arguments to consider this activation as an active contributor to tumor dissemination and growth. The role of coagulation activation in stromal remodeling is well established, e.g. the contribution of platelets in tumor angiogenesis as a major source of circulating vascular endothelial growth factor (VEGF) [8–13]. In addition, active coagulation is a prerequisite for successful tumor cell extravasation in numerous preclinical model systems and seems critical for the successful conclusion of the metastatic cascade [14, 15]. Interactions between circulating tumor cells (CTC), platelets and neutrophils including the neutrophil extracellular traps (NETs) are contributing to CTC survival and dissemination [16].

Markers of active coagulation in plasma of patients with cancer, including patients with metastatic breast cancer (MBC), enables the identification of those patients at increased risk for venous thromboembolic events (VTE) [17]. Patients with MBC and suffering from a VTE-event have a worse outcome [18]. Finally, in patients with advanced disease, a hypercoagulable state can develop into life threatening diffuse intravascular coagulation (DIC), microangiopathic thrombosis, hemolytic anemia and a combination thereof [19].

Different mechanisms have been proposed to explain this hypercoagulable state. We have previously demonstrated that venous effluent levels of plasma D-dimers in patients with localized colorectal cancer are significantly increased in comparison with arterial levels [20]. This suggests a substantial contribution of local intratumoral coagulation activation to the changes as measured in blood, suggestive of an overflow phenomenon. However, intravascular activation of the coagulation/fibrinolysis might also contribute to the overall result. The presence of tissue factor (TF) expressing circulating tumor cells (CTC) and tdEVs in the circulation is considered as one of the most important contributors to this prothrombotic activity [21].

In this study we have investigated the association between circulating tumor cells and markers of active coagulation/fibrinolysis in two cohorts of patients with MBC. The first cohort consists of patients that have participated in prior trials on CTCs in MBC and for whom, for whatever reason, coagulation tests were performed. We consider this first data set as a discovery cohort. The second cohort consists of patients with MBC that participated in the P1133 prospective study on different quantification methods for CTC enumeration.

## Methods

### Patients

#### Cohort 1

Peripheral blood samples were collected from 77 patients with MBC as part of different clinical studies exploring the prognostic role of CTC enumeration in MBC at the Sint-Augustinus Cancer Center (Antwerp, Belgium) between Jan 2001 until June 2016. The original study results have been reported in detail elsewhere [22–25]. The patients in the current analysis have been included provided coagulation tests were available within a window of 10 days from the date of the CTC enumeration. Patients were excluded if they were treated with any type of anticoagulative or anti-platelet drug or if systemic anticancer treatment was switched between CTC and coagulation sampling moments. Patients with an active or past thromboembolic event were also excluded.

#### Cohort 2

A second cohort of patients with MBC was enrolled in a prospective clinical trial at the Sint-Augustinus Cancer Center (Antwerp, Belgium) between February 2019 and December 2020. Follow-up was captured until end March 2021. This study entitled “P1133: CTC enumeration using Rarecyte and CellSearch systems: a comparative study” had as primary endpoint the comparison between CTC numeration by the CellSearch and Rarecyte platforms. All patients had either a de novo diagnosis of metastatic disease or evidence of disease progression. Patients on anticoagulation therapy or with a known other malignancy within the last 5 years were excluded. This study was approved by the GzA Ethical committee. Written informed consent was obtained from all individual participants.

### Blood sampling

Peripheral venous blood was collected using CellSave preservative (Menarini, Italy) and citrate vacutainer tubes (BD Biosciences, NJ USA) for CellSearch ® (Menarini, Italy) circulating tumor cell (CTC) analysis and platelet poor plasma preparation, respectively. Blood was collected prior to treatment for disease progression. CellSave whole blood samples were stored at room temperature until analysis.

### CTC enumeration

CellSearch® circulating tumor cell (CTC) analysis: CellSave blood samples (7.5 mL) were processed by the CellSearch® system in the Translational Cancer Research Unit (TCRU) as described elsewhere [26]. Briefly, CTCs are immunomagnetically separated from other blood components by EpCAM (epithelial cell adhesion molecule) antibody-conjugated beads and then stained for cytokeratins (CKs 8, 18 and 19), DAPI (4′,6-diamidino-2-phenylindole) and CD45 in a fluorescent-based approach. CTCs are defined as CK+DAPI+CD45− cells over 4 μm in diameter.

### Accept

Archived immunofluorescence images were retrospectively reanalyzed by means of the open source image analysis tool for Automated CTCs Classification, Enumeration and Phenotyping (ACCEPT) (https://github.com/LeonieZ/ACCEPT). Upon downloading the images into ACCEPT, the algorithm automatically provides phenotypic measurements for all detected events (larger than four pixels) per fluorescence channel. Running validated user-defined gating settings, the software was able to automatically detect and enumerate CTCs and tdEVs, and store the phenotypic characteristics of these EpCAM + entities in a ready-to-use database [26].

### Statistics

Statistical analysis GraphPad Prism software version 9.2.0 (CA, USA) was used for primary statistical analysis and graph preparation.

Continuous variables are describes by median and range.

Normality of data was confirmed by D’Agostino–Pearson normality testing for the transformed D-dim, tdEVs and CTC results.

Overall survival was measured as days from study entry till death or censored at last follow-up.

Median OS was estimated by Kaplan–Meier survival curves. Survival curves were compared with a logrank test.

## Results

### Retrospective cohort (*n* = 77)

From our CTC database with 839 patients with MBC and at least one CTC count, 77 (9.1%) patients could be identified in accordance with the limited in- and exclusion criteria. Clinical details are summarized in Table 1. Median age was 63 years (range 33–91). At the time of this analysis, all 77 patients have died from MBC. Survival is counted in elapsed days since the blood sample collection for CTC enumeration. Median overall survival is 178 days (15–3120). Although heterogeneous, this is overall a cohort with poor prognosis. Twenty five (32.5%) patients had died within three months of study entry.

**Table 1:** Retrospective patients cohort 1 (*n* = 77)

Age (median, range)	63 years (33–91)
Pathology	
IDA	55 (71.5%)
ILA	22 (28.5%)
ER+/HER2−	42 (54%)
ER−/HER2−	13 (17%)
ER−/HER2+	16 (21%)
ER+/HER2+	6 (8%)
Visceral disease	47 (61%)
Non-visceral disease	30 (39%)
Bone only	21 (27%)
Initial presentation with metastasis	38 (49%)
On adjuvant endocrine therapy	20/38
No prior chemotherapy	40 (52%)
Prior chemotherapy	37 (48%)
1 line	15
2 lines	11
3 lines	7
4 lines	2
5 lines	2
Prior trastuzumab	12

*IDA* infiltrating ductal adenocarcinoma, *ILA* infiltrating lobular carcinoma, *ER* estrogen receptor, *HER2* human epidermal growth factor receptor 2

**Fig. 1:**
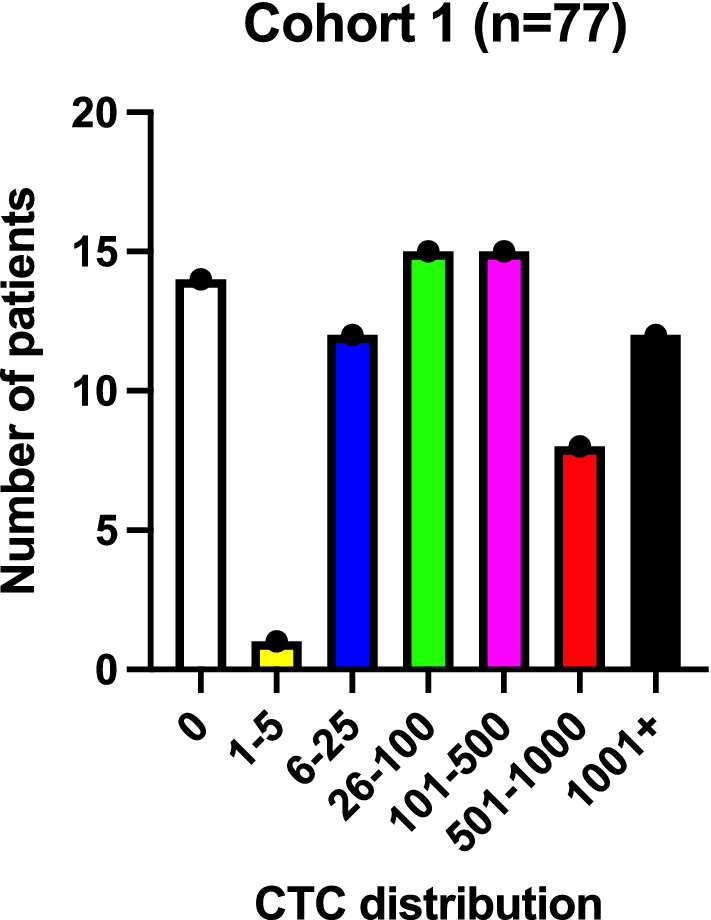
CTC count distribution cohort 1

Median CTC count was 53/7.5 mL (range 0–100,000/7.5 mL). Only 14 of the 77 patients (18%) had a CTC count lower than 5 CTC/7.5 mL. A histogram of the CTC count distribution is shown in Fig. 1.

All 77 patient had at least one routine coagulation test result outside the limits of normal.

In total 13 patients (16.8%) were diagnosed with abnormalities compatible with overt DIC.

Only 7 and 5 patients had respectively an increased activated partial thromboplastin time (aPTT) or prothrombin time (PTT). No significant association between aPTT or PTT with either CTC, platelet count or D-dimers could be identified.

#### Correlation between CTC and OS

**Fig. 2:**
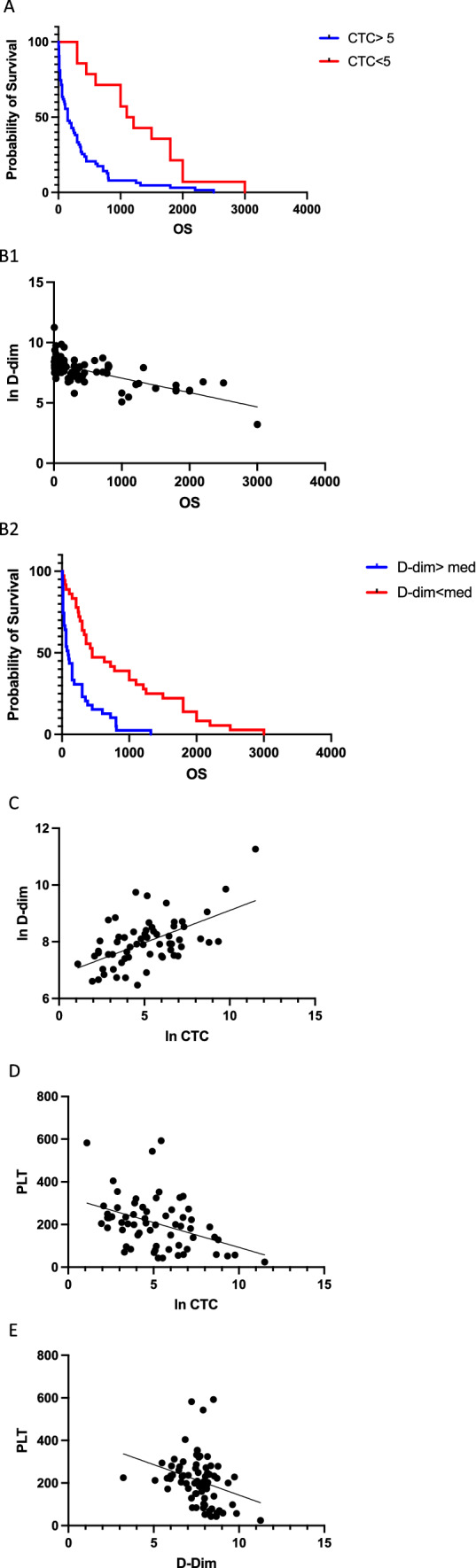
**A** Overall SURVIVAL (days) in patients with MBC for those with < 5 CTCs per 7.5 mL of whole blood and those in the group with ≥ 5 CTCs in 7.5 mL of whole blood (*n* = 77). (log rank HR 2.826, *p* < 0.0001). **B1** Relationship between the ln D-dimers (ng/mL) versus OS (days) (*R*^2^ = 0.465, *p* < 0.0001). **B2** Overall Survival (days) in Patients with MBC for those with < or > median D-dimer (*n* = 75). (log rank HR 2.586, *p* < 0.0001). **C** Relationship between ln CTC (*x*/7.5 mL) and ln D-dimers (ng/mL) (*R*^2^ = 0.3215, *p* < 0.0001). **D** Correlation between platelet count (PLT) (10^9^/mL) and the ln CTC (*x*/7.5 mL) (*p* < 0.0009, *R*^2^ 0.167). **E** Correlation between platelet count (PLT) (10^9^/mL) and the ln Dim (ng/mL) (*p* < 0.016)

The prognostic significance for overall survival of CTC count at time of progression (< or ≥ 5 CTC /7.5 mL) was confirmed (Fig. 1). Median OS was respectively 150 days versus 1155 days with a logrank HR of 2.826 (*p* < 0.0001) (Fig. 2A).

#### Correlation between D-dim and OS

The levels of D-dimer ranged between 161 and 77,735 ng/mL with a median of 2145 ng/mL. Linear regression of the ln value of D-dimers with OS was statistically significant (*p* < 0.0001, *R*^2^ = 0.4625) (Fig. 2B1).

Median OS according to the median D-dim was 93 days versus 450 days with a logrank HR of 2.586 (1.574–4.247) (*p* < 0.0001) (Fig. 2B2).

#### Correlation between CTC as a continuous variable and D-dim level

In this cohort a striking linear correlation exist between CTC enumeration and ln D-dimer level (*p* < 0.0001; *R*^2^ = 0.3215) (Fig. 2C).

#### Correlation between CTC and platelet count (PLT)

The average platelet number varied between 24 and 592 × 10^9^/mL with a median value of 206. The PLT level was correlated with the ln CTC level (*p* < 0.0009, *R*^2^ = 0.167) (Fig. 2D).

#### Correlation between D-dim and PLT

Platelet count is negatively correlated with D-dim level (*p* < 0.016) (Fig. 2E).

#### Univariate and multivariate analysis for OS

In the univariate analysis, presence of visceral disease, number of lines of chemotherapy (0 vs 1 vs 2+), ln CTC, CTC at baseline, CTC ≥ 5, the ln D-dimers, platelet count and serum LDH were significantly associated with overall survival. ER status, HER2 status, TN disease, age, and fibrinogen level were not.

In multivariate analysis only CTC ≥ 5 at baseline, ln D-dimers, lines of chemotherapy 0–1 vs 2+) and presence of visceral disease were associated with OS.

### Prospective cohort (*n* = 92)

**Fig. 3:**
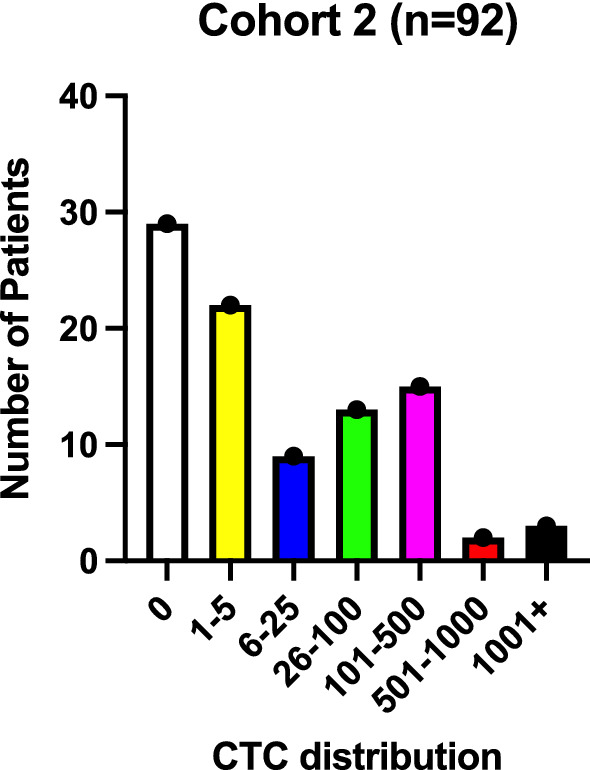
CTC count distribution cohort 2

One hundred and four patients with MBC were prospectively enrolled in a comparative CTC enumeration study called “P1133”. As one of the secondary endpoints in that study, coagulation studies (aPTT, PT, Fibrinogen and D-dimers) were systematically performed synchronous with the CTC enumeration sampling. Twelve patients with thromboembolic events and/or any type of anticoagulative therapy were excluded from the current analysis. Clinical details are summarized in Table 2. Median age was 63 years (range 34–97), nearly 4 out 5 patients suffered from ER+/HER2− disease and one in three patients presented ab initio with stage IV disease. Only 17% had received prior chemotherapy for metastatic disease. Median follow-up was 210 days (range 15–751). Median overall survival has not been reached. The CellSearch® CTC count had a median of 4 tumor cells/7.5 mL (range 0–2289). This cohort of patients had in general less advanced disease both with regard to extent of disease and number of prior treatment regimens. A histogram of the CTC count distribution is shown in Fig. 3 with only 5 patients had a CTC count in excess of 500 tumor cells/7.5 mL.

**Table 2:** prospective patients cohort 2 (*n* = 92)

Age (median, range)	63 years (34–97)
Pathology	
IDA	69 (75%)
ILA	23 (25%)
ER+/HER2−	73 (79%)
ER−/HER2−	13 (15%)
ER−/HER2+	2 (2%)
ER+/HER2+	4 (4.5%)
Visceral disease	40 (43%)
Non-visceral disease	52 (57%)
Bone only	40 (43%)
Initial presentation with metastasis	29 (31.5%)
On adjuvant endocrine therapy	10/29
No prior chemotherapy	75 (81.5%)
Prior chemotherapy	17 (18.5%)
1 line	11
2 lines	2
3 lines	2
4 lines	2
Prior trastuzumab	2

#### Correlation between CTC and OS

**Fig. 4:**
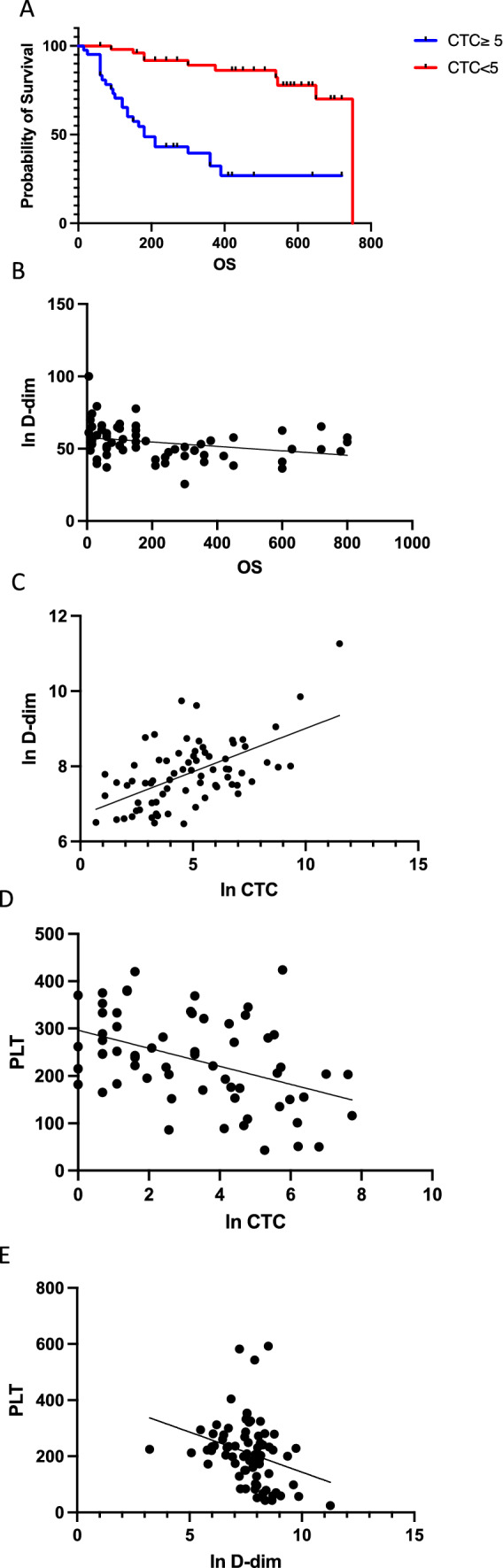
Cohort 2. **A** Kaplan–Meier estimates of OS (days) in patients with MBC for those with < 5 CTCs per 7.5 mL of whole blood and those in the group with ≥ 5 CTCs in 7.5 mL of whole blood (*n* = 92). (HR 4.167, *p* < 0.0001). **B** Relationship between the ln D-dimers (ng/mL) versus OS (days) (*n* = 84) (*R*^2^ = 0.252, *p* < 0.0001). **C** Relationship between ln CTC (*x*/7.5 mL) and ln D-dimers (ng/mL) (*n* = 92) (*R*^2^ 0.3354, *p* < 0.0001). **D** Relation between platelet count (PLT) (10^9^/mL) and the ln CTC (*x*/7.5 mL) (*n* = 92) (*R*^2^ 0.1902, *p* = 0.004). **E** Relation between platelet count (PLT) (10^9^/mL) and ln D-dimers (ng/mL) (*R*^2^ 0.1947, *p* < 0.001)

The prognostic significance of baseline CTC count (< or ≥ 5 CTC /7.5 mL) was confirmed in this cohort with a logrank HR of 4.167 (*p* < 0.0001) (Fig. 4A).

#### Correlation between ln D-dim and OS

The levels of D-dimer ranged between 100 and 30,000 ng/mL with a median of 1125 ng/mL. Increasing levels of fibrin degradation are associated with a shortened OS. (*R*^2^ = 0.252, *p* < 0.0001). Median OS differed with borderline statistical significance according to the median D-dim (135 days versus 650 days) with a logrank HR of 2.095 (0.917–4.799) (*p* = 0.068).

#### Correlation between CTC and D-dim level

Also in this cohort a striking linear correlation is observed between CTC enumeration and D-dimer level (*R*^2^ = 0.3354, *p* < 0.0001) (Fig. 4C).

#### Correlation between CTC and platelet count (PLT)

The platelet count varied between 43 and 424 × 10^9^/mL with a median value of 242. The PLT level was correlated with the ln transformed CTC level (*p* < 0.004, *R*^2^ = 0.1902) (Fig. 4D).

#### Correlation between D-dim and PLT

An inverse association between platelet count (PLT) (10^9^/mL) and ln D-dimers (ng/mL) is also present in this cohort (Fig. 4E) (*R*^2^ = 0.1947, *p* < 0.001).

#### Correlation between extracellular vesicles (ECV) and CTC

**Fig. 5:**
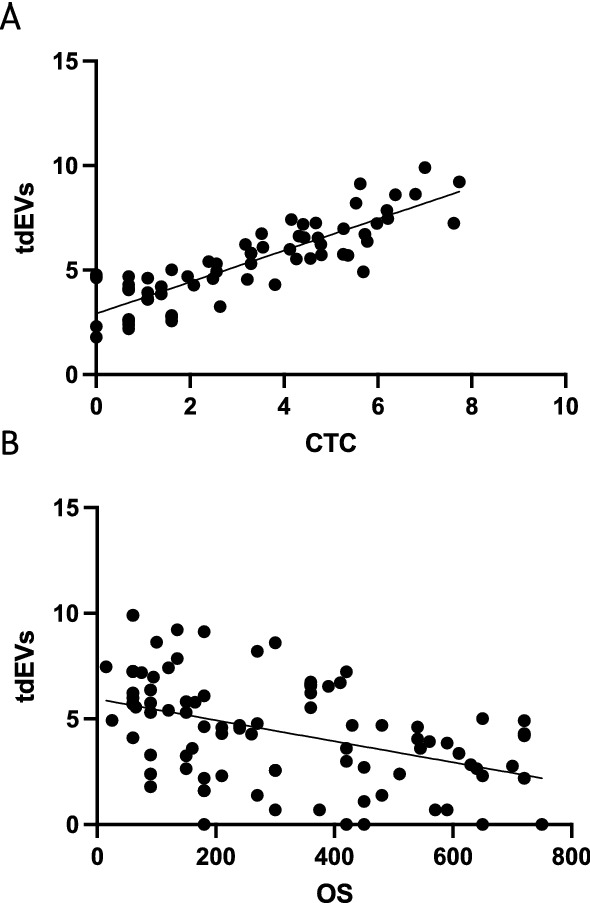
Cohort 2. **A** Regression analysis between ln CTC and ln tdEVs. (*R*^2^ 0.743, *p* < 0.0001) **B** Relation between ln tdEVs and OS (*p* < 0.001)

A significant correlation exists between ln CTC count and ln ECV with a *p* < 0.001 and *R*^2^ of 0.740. (Fig. 5A). The ECV count is also positively related to the D-dimers in an univariate analysis with *R*^2^ = 0.4160 (*p* < 0.0001). In a multiple regression analysis, relating both CTC and ECV with the D-dimers as the dependent variable, the addition of the tdEVs data failed to contribute significantly. Number of tdEVs significantly predicted OS (Fig. 5B).

#### Univariate and multivariate analysis for OS

In the univariate analysis, the performance status, visceral disease, number of lines of chemotherapy (0–1 vs 2+), CTC ≥ 5 CTC/7.5 mL, the levels of D-dimers and ECV, serum LDH, HER2 status and ER status were significantly associated with overall survival. Age, platelet count, and fibrinogen level were not.

In multivariate analysis only CTC ≥ 5 CTC /7.5 mL, ER status, HER2 status and lines of chemotherapy were associated with OS.

## Discussion

The association between cancer and thrombotic events is well established and patients with advanced breast cancer have an increased risk for venous thromboembolic events (VTE). In two cohorts of patients with stage IV breast cancer, and excluding patients with established prior or current TE events and those patients on drugs interfering with normal coagulation, the prevalence of coagulation abnormalities (defined by results crossing the boundaries of the upper limits of normality for either platelet count, D-dimers, fibrinogen, PTT and/or aPTT), was respectively 91% and 85% for the retrospective and the prospective cohort. Limiting the analysis to those patients, from both cohorts, presenting with untreated stage IV disease, did not change those frequencies. Nearly all these patients, 35 out of 37 (95%), had at least one abnormal coagulation test.

The retrospective cohort and the validation cohort both corroborate the prognostic significance of CTC ≥ 5 CTC /7.5 mL (Figs. 2A and 4A). The respective HR obtained for OS were 0.35 (0.226–0.553) and 0.123 (0.058–0.256) for patients with CTC counts less than 5. Although these two cohorts were enrolled in different time frames, the extent of disease was clearly more advanced in the retrospective cohort. In this cohort 1, 15/77 (19%) patients had 0 CTC/7.5 mL and 16/77 (20.7%) had a CTC < 5. In this cohort 1, a total 37/77 (48%) had a CTC count > 100 and eleven patients (14%) had more than 1000 CTCs/7.5 mL, suggesting that in substantial group of patients with end-stage disease, extremely elevated numbers of tumor cells are being shed into the circulation. In cohort 2, 29/92 (23%) patients had 0 CTC/7.5 mL and 47/92 (55.4%) had a CTC count < 5. These large differences in fraction of CTC positives and the high absolute numbers of CTCs in some patients in cohort 1, prompted us to opt for an additional prospectively identified confirmatory cohort of patients with MBC. The degree of CTC positivity in cohort 2 with nearly half of the patients with 5 or more CTC is clearly more in line with what has been reported previously in unselected patients with progressive MBC [25, 26].

In univariate analysis, absolute CTC count was positively associated with D-dimer levels in both cohorts. Similarly, in both cohorts, CTC numbers and D-dimer level were inversely related to platelet count. These relationships were highly significant in both cohorts. Strikingly, the positive correlation between CTC and D-dimer level was the strongest. Others have already observed a correlation between CTC positivity and increased risk for venous thromboembolism [17]. Even a positive categorical association between CTC positivity and D-dimer level has been reported [27, 28]. In both our cohorts, the positive association between CTC and D-dimer, both as a continuous variable, is an important addition to these prior observations. These results corroborate previous studies suggesting that the presence of epithelial cancer cells in the circulation acts directly is an activator of intravascular platelet and coagulation activation [29]. These results are probably an underestimation because of the exclusion of patients with a past or recent thromboembolic event and those patients deemed at a risk for such an event that it warrants drug treatment.

Tumor derived extracellular vesicles as quantified by the ACCEPT program, were significantly associated with CTC count. In univariate analysis EVC number was also associated with coagulation activation. In a multivariate analysis CTC count predominated this association both with D-dimers and platelet, and tdEVs did not add in this association.

In the 10 patients who died within 15 days after blood collection, CTC counts were very high (median count 653 CTCs/7.5 mL, mean 12,000 CTCs/7.5 mL), and platelet counts were low (median 72, mean 112) (data not shown). This suggests, accepting all the limitations of low patient numbers and case selection bias, that a “leukemic” phase with consumptive coagulation is more common than generally accepted. We have previously demonstrated in an autopsy study that a substantial fraction of CTCs is retained in the pulmonary vascular bed, and suggested, as others have done previously, that intravascular metastasis is a relevant growth model in patients with advanced disease [30, 31].

In conclusion, our study adds clinical arguments to the hypothesis for a contribution of CTCs as one mechanism of intravascular coagulation activation. We furthermore confirmed the direct association between CTC count and tdEVs. We could not confirm an independent association of these tdEVs with the D-dimer level of platelet count. The role of different types of anticoagulation have been extensively studied in patients with advanced malignancies mainly with the aim of preventing thromboembolic events. Impact of these types of anticoagulation on tumor progression is less clear. Recent studies are investigating a potentially direct antiproliferative effect of direct anticoagulants in patients with operable breast cancer [32]. This study, however lacks any mechanistic/functional analysis of the potential contribution of CTCs in the activation of coagulation. This limits any formal causal conclusion for a link between CTC numbers and coagulation disturbances.

Finally, we suggest that substantial numbers of CTCs are present in terminally ill patients with MBC.
